# Evaluation of student perceptions with 2 interprofessional assessment tools—the Collaborative Healthcare Interdisciplinary Relationship Planning instrument and the Interprofessional Attitudes Scale—following didactic and clinical learning experiences in the United States

**DOI:** 10.3352/jeehp.2019.16.35

**Published:** 2019-11-05

**Authors:** Vincent Dennis, Melissa Craft, Dale Bratzler, Melody Yozzo, Denise Bender, Christi Barbee, Stephen Neely, Margaret Robinson

**Affiliations:** 1 Department of Pharmacy, Clinical and Administrative Sciences, College of Pharmacy, University of Oklahoma, Oklahoma City, OK, USA; 2 Fran and Earl Ziegler College of Nursing, University of Oklahoma, Oklahoma City, OK, USA; 3 Department of Health Administration and Policy, College of Public Health, University of Oklahoma, Oklahoma City, OK, USA; 4 Department of Family and Preventive Medicine, College of Medicine, University of Oklahoma, Oklahoma City, OK, USA; 5 Department of Rehabilitative Sciences, College of Allied Health, University of Oklahoma, Oklahoma City, OK, USA; 6 Department of Communication Sciences and Disorders, College of Allied Health, University of Oklahoma, Oklahoma City, OK, USA; 7 Office of Instruction, Assessment, & Faculty Development, College of Pharmacy, University of Oklahoma, Oklahoma City, OK, USA; 8 Office of the Vice Provost for Academic Affairs & Faculty Development, University of Oklahoma, Oklahoma City, OK, USA; Hallym University, Korea

**Keywords:** Academic medical centers, Ambulatory care, Attitude of health personnel, Health education, Interprofessional relations, United States

## Abstract

**Purpose:**

This study investigated changes in students’ attitudes using 2 validated interprofessional survey instruments—the Collaborative Healthcare Interdisciplinary Relationship Planning (CHIRP) instrument and the Interprofessional Attitudes Scale (IPAS)—before and after didactic and clinical cohorts.

**Methods:**

Students from 7 colleges/schools participated in didactic and clinical cohorts during the 2017–2018 year. Didactic cohorts experienced 2 interactive sessions 6 months apart, while clinical cohorts experienced 4 outpatient clinical sessions once monthly. For the baseline and post-cohort assessments, 865 students were randomly assigned to complete either the 14-item CHIRP or the 27-item IPAS. The Pittman test using permutations of linear ranks was used to determine differences in the score distribution between the baseline and post-cohort assessments. Pooled results were compared for the CHIRP total score and the IPAS total and subdomain scores. For each score, 3 comparisons were made simultaneously: overall baseline versus post-didactic cohort, overall baseline versus post-clinical cohort, and post-didactic cohort versus post-clinical cohort. Alpha was adjusted to 0.0167 to account for simultaneous comparisons.

**Results:**

The baseline and post-cohort survey response rates were 62.4% and 65.9% for CHIRP and 58.7% and 58.1% for IPAS, respectively. The post-clinical cohort scores for the IPAS subdomain of teamwork, roles, and responsibilities were significantly higher than the baseline and post-didactic cohort scores. No differences were seen for the remaining IPAS subdomain scores or the CHIRP instrument total score.

**Conclusion:**

The IPAS instrument may discern changes in student attitudes in the subdomain of teamwork, roles, and responsibilities following short-term clinical experiences involving diverse interprofessional team members.

## Introduction

### Background/rationale

Health education accreditation standards continue to evolve with respect to interprofessional education (IPE) [1] through the inclusion of required experiences in medical [2] and other professional school curricula. Due to a number of identified barriers [3,4], the expectation for interprofessional practice (IPP) to manage patients with complex health conditions and social needs is limited by educational models that do not foster the team-based collaborative skills necessary to meet the inherent challenges. Even well-designed academic efforts to embed IPE in health professions programs often lack essential team-based IPP experiences. Despite these limitations, the merit of and need to advance IPE and IPP are reflected by ongoing initiatives to promote an array of methods [5] and to emphasize the need for faculty development and research to develop transferable training models that include patient-related outcomes [6]. As IPP training models evolve, it is essential to select assessment methods or tools suitable for discerning the educational impact of our interventions, although guidance is currently limited [7].

### Objectives

This study aimed to describe our experiences with the design and delivery of interprofessional learning experiences to didactic (n=865) and clinical (n=76) student cohorts at our comprehensive academic health center. We compared 2 validated survey instruments using a randomized design before and after the interprofessional learning experiences. Our specific objectives included assessing students’ perceptions of their interprofessional experiences to determine the impact of both modalities and sharing the details of our educational interventions for replication and refinement. The hypothesis was that brief longitudinal clinical experiences in IPP (the clinical cohort) would produce larger changes in student attitudes than didactic small-group interactive IPE experiences (the didactic cohort).

## Methods

### Ethics statement

This research received an exemption approval from the campus Institutional Review Board for the Protection of Human Subjects and met the criteria for a waiver of informed consent (IRB #8434).

### Study design

A randomized parallel cohort study of 2 validated survey instruments was deployed prior to and after didactic interactive small-group (All Professions Day [APD] 1 and 2) and clinical cohort (Empowering Patients through Interprofessional Collaboration, EPIC) learning experiences to measure changes in students’ perceptions according to their learning modality and assigned assessment instrument. The descriptions in the text follow the STROBE statement (https://strobe-statement.org/).

### Participants

The participants were students enrolled in the Colleges of Allied Health, Dentistry, Medicine, Nursing, Pharmacy, Public Health, and the School of Social Work at the University of Oklahoma in the southern United States during the 2017–18 academic year. For the APD cohort, a total of 865 students from 25 degree programs at 7 colleges participated on 1 of 108 teams of 8 to 10 students. Each APD team was facilitated by 1 of 51 trained faculty members. All interprofessional student programming was intentionally based on the Interprofessional Education Collaborative Competencies (IPEC) [8,9]. The faculty engaged the APD cohort in a mixture of lecture, active learning, and team-building activities to introduce the 4 IPEC competencies and to achieve the learning objectives between the fall and spring semesters (Table 1). Parallel to the APD cohort, a separate cohort of 76 advanced career students representing 14 professional programs was assigned to complete an IPP clinical experience (EPIC) at a local charitable outpatient clinic after receiving 2 separate preparatory sessions.

### Setting

The orientation (the first preparatory session) was 4 hours in length and included a team-building activity, an introduction to complex patient care in the context of social determinants of health, and a team discussion to develop a clinical strategy to coordinate/deliver care. The second preparatory session (4 hours in length) provided an orientation to the outpatient clinic, a structured clinical simulation using standardized patients to test and refine the teams’ strategy to coordinate care delivery when attending their clinical sessions, and an orientation to the web-based electronic medical record. After the 2 preparatory sessions, the student teams were scheduled to provide care at a charitable clinic each week for 4 hours on Thursday evening. Each of the 8 teams hosted a clinical session once monthly, with 2 teams per night. They repeated the sequence until all teams had completed 4 clinical sessions, approximately once monthly over 4 months during the fall and spring semesters. Two initial patients with complex medical and social needs were scheduled per team on the first clinic night, with 2 to 3 patients scheduled as a mixture of new and/or follow-up patients on subsequent clinic nights. A faculty facilitator drawn from any discipline and an attending medical provider were scheduled per team, with additional faculty members scheduled according to regulatory supervision requirements for the participating disciplines. The personnel of the charitable clinic were compensated for managing duties including scheduling and rooming patients, and a pharmacist was also compensated for fulfilling prescription orders during the evening clinics. Access to the web-based electronic medical record was granted to allow students and faculty to document their clinical encounters. Patient care was coordinated and delivered by the interprofessional student team members, according to their IPP team strategy, which was modified as required based on the needs of the patient. The clinical encounter time per patient was structured with approximately 45 minutes for patient assessment, 15 minutes for presentation/discussion with the attending medical provider/faculty member(s), and 15 minutes for the final interaction with the patient prior to scheduling follow-up actions. The student teams worked with patients to set goals at each encounter and modified the plan longitudinally as needed on subsequent visits. Final documentation in the electronic medical record was co-signed by the attending medical provider. A debriefing session of approximately 15 minutes was guided by the designated team non-medical provider faculty facilitator and concluded the first 3 of the 4 clinic sessions to prompt reflective learning and planned adjustments to care delivery in subsequent clinical sessions. The fourth and final clinic session was concluded by a global reflection on the interprofessional learning experience with an open discussion of 8 questions and response summaries by the faculty facilitators.

### Variables and measurements

Two validated survey instruments were used, with permission of the authors, to assess changes in student perceptions. Students were requested to complete the instrument prior to the first planned event and following the last planned event, according to their assigned cohort. The Interprofessional Attitude Scale (IPAS) is a 27-item instrument that assesses the 5 subdomains of teamwork, roles, and responsibilities (9 questions), patient centeredness (5 questions), interprofessional biases (3 questions), diversity and ethics (4 questions), and community centeredness (6 questions) [10]. The Likert scale was scored as follows: 1, strongly disagree; 2, disagree; 3, somewhat disagree; 4, neither agree nor disagree; 5, somewhat agree; 6, agree; and 7, strongly agree. According to the survey instructions, 1 question was reverse scored. The total score and sub-domain averages were calculated.

The Collaborative Healthcare Interdisciplinary Relationship Planning (CHIRP) instrument, included in Supplement 1, is a 14-item survey that assesses a single domain, attitudes toward interdisciplinary teamwork [11]. Both surveys were created separately and administered via the online survey tool Qualtrics (https://www.qualtrics.com/, Provo, UT, USA). The IPAS survey had total of 49 questions that included 4 academic questions, the 27 validated IPAS questions, and 18 additional experimental IPAS questions that are undergoing validation (Supplement 2). The CHIRP survey had 18 questions (14 validated survey items and 4 academic questions). A 5-point scale of agreement was used and scored as follows: 1, I do not agree at all; 2, I somewhat agree; 3, I fairly much agree; 4, I very much agree; 5, I completely agree. The average was calculated as the overall score.

### Procedure

The baseline IPAS and CHIRP surveys were sent to 429 and 436 students, respectively. For each survey, 18% of respondents were from allied health professions, 12% from dentistry, 28% from medicine, 16% from nursing, 11% from pharmacy, 4% from public health, and 10% from social work. The overall baseline response rate was 58.7% for the IPAS and 62.4% for the CHIRP survey. After accounting for dropout, the post-2nd APD IPAS survey was sent to 348 students, of whom 54.0% responded. Similarly, the post-2nd APD CHIRP survey was sent to 353 students and 61.8% responded. Post-EPIC IPAS surveys were sent to 36 students, with a response rate of 97.2%. Lastly, post-EPIC CHIRP surveys were sent to 37 students and the response rate was 105.4%; we believe this error was due to some respondents misinterpreting the EPIC participation question as indicating their interest in future participation instead of actual participation during the study period.

### Technical information

Students were randomly assigned to complete 1 of the 2 survey instruments in a manner that ensured an even distribution of disciplines across both survey instruments. Survey responses were anonymous in accordance with IRB approval. Due to the anonymity of the responses, baseline and post-intervention data could not be linked to individual participants; therefore, traditional longitudinal analysis techniques could not be employed. The results were pooled and presented as overall baseline, post-2nd APD, and post-EPIC clinic results. Descriptive statistics were utilized to summarize the response rates and survey results. The response rates are reported as frequency (percent), while the survey results are presented as mean (standard deviation), minimum, median, and maximum values (Datasets 1, 2).

### Bias

The impact of non-responders was of primary concern, and in accordance with published best practice guidelines [12] we sought to reach at least a 50% response rate. Additionally, responses were anonymous to address the potential impact of social desirability bias.

### Study size

An a priori sample size calculation was not performed due to the large (n=865), campus-wide IPE effort to which our study was attached.

### Statistical methods

The Pittman nonparametric test using permutations of linear ranks was used to determine any differences in the distribution of scores. Due to the overall large sample size, exact permutation tests were not computationally possible; therefore, Monte Carlo estimates of the test’s P-value were used. Each test was set to run for 50,000,000 permuted samples, which resulted in 99% confidence intervals (CIs) to the ten-thousandths around the P-value (e.g., estimated P-value=0.0673; 99% CI, 0.0672–0.0674).

Group comparisons were made for the CHIRP overall score, IPAS total score, and each IPAS subdomain score. For each of these scores, 3 comparisons were made simultaneously: (1) overall baseline versus post-2nd APD; (2) overall baseline versus post-EPIC clinic; and (3) post-2nd APD versus post-EPIC clinic. To account for the simultaneous comparisons, alpha was adjusted to 0.0167 using the Bonferroni method. All tests were 1-tailed with the hypotheses that overall baseline score < post-2nd APD score < post-EPIC clinic score. All analyses were conducted using SAS software ver. 9.4 (SAS Institute Inc., Cary, NC, USA).

## Results

The pooled results for the IPAS survey are reported in Table 2. Collectively, students responded with high levels of agreement at baseline. The lowest score was the interprofessional bias subdomain, with a median of 5.0, and the highest were the patient centeredness subdomain and the diversity and ethics subdomain, each of which had a median of 6.8. The median scores moved slightly upwards for the post-2nd APD scores, but none were significantly different relative to baseline. For students participating in the EPIC clinic cohort, the teamwork, roles, and responsibilities subdomain was found to be significantly higher than both the overall baseline scores and the post-2nd APD scores. No other comparisons were found to yield significant differences.

Table 3 reports the pooled results for the CHIRP survey. No significant differences were found between groups. A *post hoc* exploratory analysis found that individual questions differed between the baseline and post-course evaluations, but the data are not reported here.

## Discussion

### Key results

Our findings suggest that a series of 4 interprofessional ambulatory clinic session experiences following 2 preparatory sessions could significantly improve students’ perceptions as assessed by the IPAS instrument. The results were strongly influenced by improved responses in the IPAS teamwork, roles, and responsibilities subdomain. Purely didactic active learning activities can provide foundational principles for IPP, but application in the context of actual patient care is expected to provide more measurable changes in student perceptions, which the IPAS results did reveal in the aforementioned subdomain. Since the clinic design provides only 4 sessions of 3–4 hours per team, this subdomain may be most sensitive to changes in response to time-limited IPP. To our knowledge, this is the first report using the validated IPAS instrument to assess all health disciplines following delivery of actual patient care as an interprofessional team among students enrolled in medicine, nursing, pharmacy, physician assistant, public health, and allied health programs [10].

### Links to other reports

Our results are consistent with a report that used IPAS prior to and following a 1-month psychiatry clerkship involving students from medicine, physician assistant, pharmacy, and social work programs [13]. Following the psychiatry clerkship, a statistically significant increase (P=0.036) was seen only in the subdomain of teamwork, roles, and responsibilities for the 59 students completing the survey. This IPAS subdomain contains 9 of the 27 survey items and includes several that frame shared learning experiences in terms of individual and team benefits. In contrast to the IPAS results, the CHIRP instrument did not show a significant overall difference in either interprofessional intervention group. The CHIRP survey was selected as a second instrument based on its previous use in multiple methods of learning including lecture instruction, interactive videos with audience response, group role-play exercises, and group high-fidelity simulations among medical and nursing students [14]. However, the CHIRP instrument was originally validated only among medical and nursing students [11], and to our knowledge it has not been deployed at a comprehensive academic health center involving all student disciplines, nor involving students providing direct patient care, so it is unclear whether these variables impacted the ability of the instrument to discern any differences in our diverse study population.

### Limitations

The limitations of our results should be acknowledged, including the lack of a control group, anonymous survey completion limiting paired data analysis, differing general experience levels between the didactic and clinical cohorts, discrepancies in the length of the survey (CHIRP versus IPAS), and involvement of students from a single institution. The finding that the post-EPIC response rate slightly exceeded the expected response rate for the CHIRP survey also suggests a small number of students did not categorize their participation in the interprofessional clinical experience accurately, having only participated in the didactic/interactive APD experiences. Such occurrences would most likely decrease the post-EPIC scores, but were infrequent enough that the effect on the final CHIRP scores would be minimal.

### Interpretation

A strength of the results for the EPIC clinical experience is that measurements were made across 8 separate interprofessional team groups consisting of 8–10 students per team, which may have facilitated homogeneity of the responses to the teamwork, roles, and responsibilities subdomain of the IPAS after only 4 ambulatory care patient clinic sessions. With respect to the CHIRP survey being significantly shorter than the IPAS survey, the potential exists for longer surveys to require extended concentration. However, only the first 31 questions of the IPAS were inclusive of the demographic and validated items, which compares more favorably to the 18 analogous items of the CHIRP. We suggest that the primary risk with the additional 18 pilot questions for the IPAS is for students to abandon completion of the survey, which may have occurred as shown by a lower percent completion for the IPAS. However, we only analyzed completed IPAS surveys, so we believe that those who persisted would be able to register meaningful responses. Although the EPIC cohort did have a generally higher experience level than the didactic cohort, we believe that the degree of change in student responses pre- and post-cohort would be more dependent on the nature of the interprofessional learning experience than on pre-existing general or interprofessional experience.

Overall, we believe our efforts represent broad inclusion of all professions at a comprehensive academic health center, as well as social work, and survey completion rates that are generally representative. The results of our educational interventions support a modification in student attitudes/perceptions consistent with level 2a of Kirkpatrick’s hierarchy (KH), which has been used to evaluate the effectiveness of training programs [15,16]; ultimately, the impact of interprofessional learning experiences should progress to higher levels of KH, including modification of knowledge/skills, behavior change, and improvement of patient outcomes. Future considerations for our curriculum design and assessment include ensuring APD participation by all early-career learners in our collective college curricula and exploring additional interprofessional patient care assessment instruments/methods to move beyond the modification of student perceptions to capture observable elements of student IPP competency.

### Conclusion

Since the post-EPIC clinic team subdomain survey score was significantly higher than the overall baseline and post-didactic cohort scores, our hypothesis that brief longitudinal ambulatory clinic experiences in IPP would produce larger changes in student perceptions than didactic small group interactive IPE experiences was accurate. The IPAS instrument may be useful for detecting modifications of student attitudes in the teamwork, roles, and responsibilities domain in response actual IPP experiences, and it has now been studied in team-based ambulatory care involving learners from a broad range of health disciplines.

## Figures and Tables

**Table 1.** APD didactic curriculum

Session	Objectives/IPEC core competencies	Activity/format (min)
Fall semester (APD1)	1. Communicate one’s roles and responsibilities clearly to families, community members, and other professionals (RR1)	Speed dating with 6 question prompts (30)
	2. Communicate information clearly to other professionals in a manner that is not discipline-specific (CC2)	Live patient case with team discussion, prioritization of concerns/profession(s) to address as a team and roaming microphone de-brief (45)
Spring semester (APD2)	(Review fall RR1 and CC2 competencies)	Second date with 8 question/reflection prompts (20)
	1. Increase understanding of the value of interprofessional collaboration as an approach to efficiently and effectively achieve a planned outcome (teamwork)	Team activity creating a U.S. map from various diagram stages with de-briefing of the process and an overhead presentation (25)
	2. Respect the unique differences in culture, values, roles and responsibilities, and expertise of other health professions (VE4)	Dyad compare/contrast of professional codes of ethics with small group sharing (20)
	3. Engage other health professionals, appropriate to the specific care situation, in shared patient-centered problem-solving (TT3)	Patient safety case study and video with fatal safety error and team failure; group discussion and consensus answers to 3 audience response questions addressing reasons for team failures, responsibilities for engagement and active roles of all members (including the patient/family members) (25)
	4. Integrate the knowledge and experience of other professions to inform care decisions, while respecting patient and community values and priorities or preferences for care (TT4)	

APD, All Professions Day; IPEC, Interprofessional Education Collaborative; RR, roles/responsibilities; CC, communication; VE, values and ethics; TT, teamwork.

**Table 2.** Pooled baseline and post-cohort results of the IPAS

IPAS^a)^	Pre-cohort overall (n=252)		Post-cohort			
			APD only (n=188)		EPIC clinic (n=35)	
	Mean±SD	Min, median, max	Mean±SD	Min, median, max	Mean±SD	Min, median, max
Teamwork, roles, and responsibilities	5.9±0.8	1.7, 6.0, 7.0	6.1±1.0	1.7, 6.2, 7.0	6.4±0.7^b),c)^	4.0, 6.8, 7.0
Patient centeredness	6.6±0.7	1.0, 6.8, 7.0	6.7±0.7	1.0, 7.0, 7.0	6.7±0.6	4.2, 7.0, 7.0
Interprofessional bias	4.8±1.1	1.0, 5.0, 7.0	4.8±1.3	1.0, 5.0, 7.0	4.9±1.1	2.7, 5.0, 7.0
Diversity and ethics	6.6±0.7	1.0, 6.8, 7.0	6.6±0.7	1.0, 7.0, 7.0	6.7±0.7	4.3, 7.0, 7.0
Community centeredness	6.2±0.7	1.0, 6.3, 7.0	6.4±0.8	1.0, 6.5, 7.0	6.4±0.8	3.7, 6.7, 7.0
Total score	6.0±0.6	1.0, 6.1, 7.0	6.1±0.7	1.0, 6.2, 7.0	6.2±0.6	4.1, 6.4, 7.0

IPAS, Interprofessional Attitudes Scale; APD, All Professions Day; EPIC, Empowering Patients through Interprofessional Collaboration, SD, standard deviation.

a) Seven-point Likert scale of agreement with 1, strongly disagree; 2, disagree; 3, somewhat disagree; 4, neither agree nor disagree; 5, somewhat agree; 6, agree; and 7, strongly agree.

b) Post-EPIC clinic significantly different from overall baseline.

c) Post-EPIC clinic significantly different from post-2nd APD.

**Table 3.** Pooled baseline and post-cohort results of the CHIRP scale

CHIRP^a)^	Pre-cohort overall (n=272)		Post-cohort			
			APD only (n=218)		EPIC clinic (n=39)	
	Mean±SD	Min, median, max	Mean±SD	Min, median, max	Mean±SD	Min, median, max
Overall score	4.0±0.5	2.1, 4.0, 5.0	4.0±0.5	2.4, 4.0, 5.0	4.2±0.7	1.0, 4.2, 5.0

CHIRP, Collaborative Healthcare Interdisciplinary Relationship Planning; APD, All Professions Day; EPIC, Empowering Patients through Interprofessional Collaboration; SD, standard deviation.

a) Five-point scale of agreement with 1, I do not agree at all; 2, I somewhat agree; 3, I fairly much agree; 4, I very much agree, 5, I completely agree.

## Footnotes

**Data availability** Data files are available from https://doi.org/10.7910/DVN/GL0RPU Dataset 1. CHIRP responses. Dataset 2. IPAS responses.

## Supplementary materials

Data files are available from https://doi.org/10.7910/DVN/GL0RPU

Supplement 1. Collaborative Healthcare Interdisciplinary Planning Scale (CHIRP).

Supplement 2. Interprofessional Attitudes Scale (IPAS).

Supplement 3. Audio recording of the abstract.
