# Supplementary material for: Evaluation of student perceptions with 2 interprofessional assessment tools—the Collaborative Healthcare Interdisciplinary Relationship Planning instrument and the Interprofessional Attitudes Scale—following didactic and clinical learning experiences in the United States
Source: J Educ Eval Health Prof. 2019 Nov 5;16:35. doi: 10.3352/jeehp.2019.16.35 (PMC6881189; doi:10.3352/jeehp.2019.16.35)
Supplement: Supplementary file 3 — Supplement 1. Collaborative Healthcare Interdisciplinary Planning Scale (CHIRP). [file jeehp-16-35-suppl1.pdf]

Collaborative Healthcare Interdisciplinary Planning Scale (CHIRP)

Item[A]	Statement
C1	A health care team works best when one or two professionals are the recognized team leaders.
C2	Optimum patient care requires that the observations of every health care professional serving a patient be included in the patient's treatment.
C4	It is often the case that health care professionals from other disciplines will have knowledge of patient care issues that I may not have considered
C6	I must consider the interest of every professional, patent and family member involved in a medical decision.
C14	Each member of a health care team should provide equal input into a patient's care.
C15	Pharmacists, nurses, physicians, social workers and other health care professionals are of equal importance in providing patient care.
C17	It is my responsibility to ensure that every member of the health care team has the opportunity to provide their input into patent care decision making.
C21	My healthcare organization/school expects me to assert myself during patient care.
C23	It is possible that a person from another healthcare discipline could have a better understanding of a patient's condition or treatment than I do.
C25	It is important that all healthcare decisions be made following a chain of command.
C27	I need the expertise of health care professionals from other disciplines to provide patient care.
C29	I feel confident in my knowledge and am willing to share my ideas with members of a health care team.
C31	I do not mind openly discussing my ideas on patient care with other healthcare professionals.
C36	I enjoy volunteering my ideas and expertise to group projects.

[A]Item designation of the 14 fitting items for the validated teamwork attitudes scale [11]

Likert scale responses (1) I do not agree at all, (2) I somewhat agree, (3) I fairly much agree, (4) I very much agree, (5) I completely agree

Reference 11. Hollar D, Hobgood C, Foster B, Aleman M, Sawning, S. Concurrent validation of CHIRP, a new instrument for measuring healthcare student attitudes toward interdisciplinary teamwork. *J Appl Measure* 2012;13:360-375.
