# Supplementary material for: Evaluation of student perceptions with 2 interprofessional assessment tools—the Collaborative Healthcare Interdisciplinary Relationship Planning instrument and the Interprofessional Attitudes Scale—following didactic and clinical learning experiences in the United States
Source: J Educ Eval Health Prof. 2019 Nov 5;16:35. doi: 10.3352/jeehp.2019.16.35 (PMC6881189; doi:10.3352/jeehp.2019.16.35)
Supplement: Supplementary file 4 — Supplement 2. Interprofessional Attitudes Scale (IPAS). [file jeehp-16-35-suppl2.pdf]

Interprofessional Attitudes Scale (IPAS)

Scale with Sub-Scales:

1. Teamwork, Roles, and Responsibilities
   - 1.1. Shared learning before graduation will help me become a better team worker.
   - 1.2. Shared learning will help me think positively about other professionals.
   - 1.3. Learning with other students will help me become a more effective member of a health care team.
   - 1.4. Shared learning with other health sciences students will increase my ability to understand clinical problems.
   - 1.5. Patients would ultimately benefit if health sciences students worked together to solve patient problems.
   - 1.6. Shared learning with other health sciences students will help me communicate better with patients and other professionals.
   - 1.7. I would welcome the opportunity to work on small-group projects with other health sciences students.
   - 1.8. It is not necessary for health sciences students to learn together.
   - 1.9. Shared learning will help me understand my own limitations.

2. Patient-Centeredness
   - 2.1. Establishing trust with my patients is important to me.
   - 2.2. It is important for me to communicate compassion to my patients.
   - 2.3. Thinking about the patient as a person is important in getting treatment right.
   - 2.4. In my profession, one needs skills in interacting and co-operating with patients.
   - 2.5. It is important for me to understand the patient's side of the problem.

3. Interprofessional Biases
   - 3.1. Health professionals/students from other disciplines have prejudices or make assumptions about me because of the discipline I am studying.
   - 3.2. I have prejudices or make assumptions about health professionals/students from other disciplines.
   - 3.3. Prejudices and assumptions about health professionals from other disciplines get in the way of delivery of health care.

4. Diversity & Ethics

   It is important for health professionals to:
   - 4.1. Respect the unique cultures, values, roles/responsibilities, and expertise of other health professions.
   - 4.2. Understand what it takes to effectively communicate across cultures.
   - 4.3. Respect the dignity and privacy of patients while maintaining confidentiality in the delivery of team-based care.
   - 4.4. Provide excellent treatment to patients regardless of their background (e.g. race, ethnicity, gender, sexual orientation, religion, class, national origin, immigration status, or ability).

5. Community-Centeredness

   It is important for health professionals to:
   - 5.1. Work with public health administrators and policy makers to improve delivery of health care.
   - 5.2. Work on projects to promote community and public health.
   - 5.3. Work with legislators to develop laws, regulations, and policies that improve health care.
   - 5.4. Work with non-clinicians to deliver more effective health care.
   - 5.5. Focus on populations and communities, in addition to individual patients, to deliver effective health care.
   - 5.6. Be advocates for the health of patients and communities.

Likert scale responses (1) Strongly disagree, (2) Disagree, (3) Somewhat disagree, (4) Neither agree nor disagree, (5) somewhat agree, (6) Agree, (7) Strongly agree

Reference 10. Norris J, Carpenter JG, Eaton J, et al. Development and construct validation of the interprofessional attitudes scale. *Acad Med* 2015;90:1-17. DOI:10.1097/ACM.0000000000000764
